# Bovine tuberculosis in Spain, is it really the final countdown?

**DOI:** 10.1186/s13620-023-00241-0

**Published:** 2023-07-25

**Authors:** Javier Bezos, José Luis Sáez-Llorente, Julio Álvarez, Beatriz Romero, Alberto Díez-Guerrier, Lucas Domínguez, Lucía de Juan

**Affiliations:** 1 Departamento de Sanidad Animal, Facultad de Veterinaria, Universidad Complutense de Madrid, Madrid, Spain; 2 VISAVET Health Surveillance Centre, Complutense University of Madrid, Madrid, Spain; 3 Ministerio de Agricultura, Pesca y Alimentación, Madrid, Spain

**Keywords:** Tuberculosis, Eradication, Control, Spain, Epidemiology

## Abstract

Bovine tuberculosis (bTB) is a severe zoonotic disease that has major impacts on both health and the economy, and which has been subjected to specific eradication programmes in many countries for decades. This manuscript highlights the relevance of this disease in the context of the European Union (EU) and summarizes the epidemiological situation and the main tools (e.g. antemortem diagnostic tests, slaughterhouse surveillance, laboratories, comprehensive databases, etc.) used to control and eradicate bTB in the various EU countries with a focus on the situation in Spain. A comprehensive description of the specific bTB epidemiological situation in Spain is provided, together with an assessment of the evolution of different epidemiological indicators throughout the last decades. Moreover, the main features of the Spanish bTB eradication programme and its control tools are described, along with the studies carried out in Spain that have allowed the updating of and improvement to the programme over the years with the aim of eradication, which has been established for 2030.

## Tuberculosis as a zoonosis

According to data published by the WHO, it is estimated that more than 140,000 people become ill and more than 12,000 people die each year as a result of zoonotic TB (zTB, i.e. tuberculosis caused by members of the *Mycobacterium tuberculosis* complex (MTBC), mainly *Mycobacterium bovis*, from animal origin), especially in Africa and Southeast Asia. As an example, in 2016 there were 147,000 new cases of zTB in the world, and 12,500 people died from it [1]. This signifies that, zTB is a serious threat to public health. However, estimates of the global burden of zTB are imprecise owing to the scarcity of reliable data caused by a lack of health surveillance in several countries, especially in those in which the infection in cattle (bovine TB, bTB) is endemic and the laboratory capacity to isolate and differentiate *Mycobacterium bovis* from *M. tuberculosis* is limited or even absent. An accurate estimate of the importance of zTB is impossible in those mostly low and middle income countries, thus making it a clearly underestimated disease [2].

The control and eradication of TB infection in cattle through the use of diagnosis and cull strategies continues to be the mainstay as regards reducing the risk of zTB. Given the high burden of human TB (caused by *M. tuberculosis*) in endemic areas, animals can also be affected by reverse zoonosis, including multi-drug resistant strains [3].

According to the European Food Safety Authority (EFSA), 111 confirmed cases of human TB resulting from *M. bovis *or *M. caprae* were reported in the EU in 2021 (32 of them in Spain). The EU notification rate in 2021 was 0.03 cases per 100,000 population members, which was identical to that reported in 2020. This EU notification rate decreased by 28.3% and 25.3%, respectively (i.e. with or without taking the data concerning the UK into account), when compared with the mean rate observed during the pre-COVID pandemic years from 2017 to 2019. Interestingly, *M. bovis* and *M. caprae* notification rates did not differ significantly between EU countries with a disease-free status as regards cattle and those with a non-disease-free status [4].

## Situation of bovine TB in Europe and Spain

In the EU, the situation regarding bTB is heterogeneous and has improved in recent years, although not significantly. The strategy employed to eradicate bTB includes a variety of measures. At the EU level, the Subgroup of the Bovine TB Task Force (Task Force Subgroup DG SANTE) has provided reports prepared by groups of experts in the field. Of these, the document “Working Document on Eradication of bovine TB in the EU” is worth highlighting, since it establishes specific guidelines that Member States should follow in order to accelerate the eradication process (SANCO/10,067/2013). Infection with *M. tuberculosis* complex (*M. bovis, M. caprae *and *M. tuberculosis*) in bovine animals and other mammalian species are included in the list of notifiable animal diseases (Commission Implementing Regulation (EU) 2018/1629) of Regulation (EU) 2016/429 on transmisible animal diseases (“Animal Health Law”) that was adopted in March 2016. Commission Delegated Regulations (EU) 2020/688 (animal health requirements for movements within the Union of terrestrial animals and hatching eggs) and 2020/689 (rules for surveillance, eradication programmes, and disease-free status for certain listed and emerging diseases) have suplemented Regulation (EU) 2016/429 and replaced Council Directive 64/432/ECC. The eradication of bTB has been an important issue since the beginnings of the European Economic Community (EEC) in 1957, and current EU policies on the eradication of the disease are best understood after considering the progressive development of relevant legislation.

The scientific report published annually by EFSA and the European Centre for Disease Prevention and Control (ECDC) on the situation of zoonotic diseases in Europe [4] provides regular updates on the situation regarding bTB in Europe. In the last 10 years (2012–2021), the overall number of cattle herds infected annually that is reported in non-disease-free zones decreased by 47.5%, whereas prevalence decreased by only 4.2%. These differences could be attributed to (i) the withdrawal of the United Kingdom from the EU; (ii) a decrease in the number of herds; (iii) the gradual attainment of a disease-free status in areas in non-disease-free Member States (MSs), and (iiii) unfavourable environmental conditions hindering the eradication process in several non-disease-free areas [4].

In 2021, the overall proportion of cattle herds testing positive for bTB was very low (9,690 out of 1,726,451 herds; 0.6%), but slightly higher than that reported in 2020 (0.4%). Several MSs did not report cases of bTB (Austria, Belgium, Czechia, Denmark, Estonia, Finland, France, Germany, Hungary Latvia, Lithuania, Luxembourg, the Netherlands, Poland, Slovakia, Slovenia and Sweden), whereas other MSs (Bulgaria, Croatia, Cyprus, Greece, Ireland, Italy, Malta, Portugal, Romania, Spain and the United Kingdom-Northern Ireland) reported the presence of bTB in their territory. The overall bTB herd prevalence was 1.3%. When comparing the data for 2021 with those for 2020, the overall number of cattle herds infected annually, the prevalence and the total number of cattle herds increased in these non-disease-free zones. This increase was mainly attributed to the withdrawal of the United Kingdom from the EU in 2020 and to the subsequent (2021) inclusion of data concerning Northern Ireland (UK), a region with a bTB prevalence that is relatively higher than in the UK as whole. The prevalence of bTB in 2021 varied widely among those MSs with a non-disease-free status: Ireland (4.6%) and Spain (1.3%) were the only MSs to report a prevalence higher than 1%. Northern Ireland, which has no disease-free zones, reported a prevalence of 11.3% [4].

Most of the MSs reported MTBC infections in 2021 without specifying the species involved. Infections with *M. bovis* were reported in Bulgaria, France, Germany, Hungary, Ireland, Italy, Poland, Romania and the United Kingdom (Northern Ireland), whereas infections owing to *M. caprae* were reported by Austria, Germany and Romania. No cases of *M. tuberculosis* infection in cattle were reported [4].

**Fig. 1:**
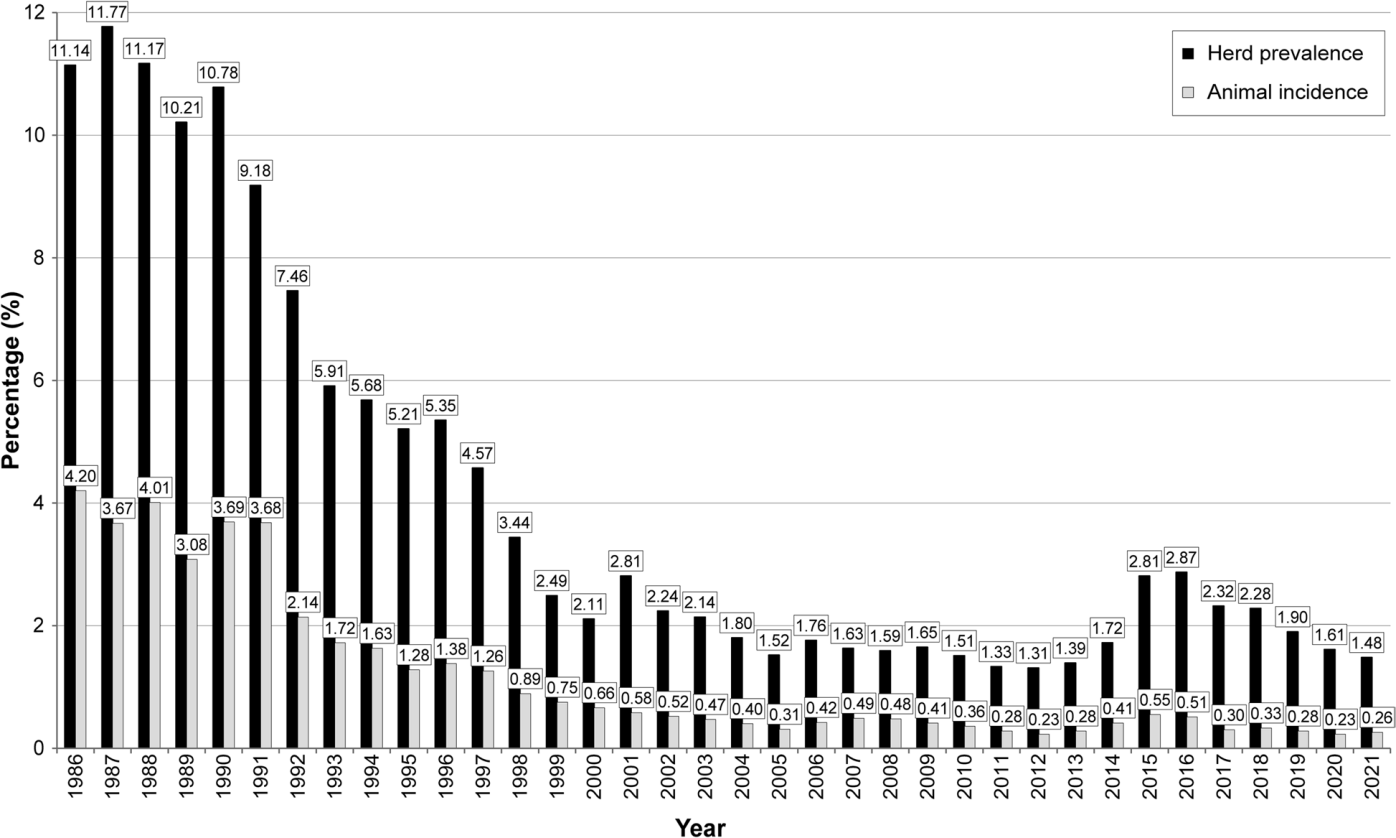
Herd prevalence and animal incidence of bovine tuberculosis in Spain in the period 1986–2021. (Data provided by the Spanish Ministry of Agriculture, Fisheries and Food, 2022)

In Spain, herd prevalence had undergone a moderate decrease in the 15 years prior to 2013, after which this indicator increased, especially in 2015 and 2016, reaching a level similar to that reported in 2001 (Fig. 1). In 2017 there was a significant decrease in prevelance of 19% when compared to 2016, and of 1.7% (not significant) in 2018 when compared to 2017. There were further decreases of around 17% in 2019 and 2020. With regard to herd incidence in the period 2003–2020, there was an overall increasing trend from 2013 on, reaching a maximum value in 2015 (2.02%) and subsequently decreasing to values similar to those reported 20 years ago. Fortunately, the incidence reported in 2021 was significantly better than that of previous years (Fig. 1). According to this data, it can be concluded that, after an increase in the epidemiological indicators (probably owing to an increase in the field diagnostic performance as a consequence of the training courses established in 2012 for all veterinarians involved in the eradication programme and the subsequent on-the-spot controls carried out by the Spanish Official Veterinary Services as regards the skin test performance), a decreasing trend began in 2016 [5].

## The epidemiology of bovine tuberculosis in Spain

The complete eradication of bTB is difficult to achieve not only in high prevalence regions but also in those with low proportions of TB-infected herds. Cattle are considered the most important host (mainly beef and bullfighting cattle), but the disease has been reported in a wide range of other domestic and wildlife species on the Iberian Peninsula that can act as reservoirs [6, 7]. In Spain, there is evidence that small ruminants, extensively-farmed pigs, wild boar and red deer may play a role in the maintenance of the disease under specific epidemiological circumstances [8–12], especially in regions in which the populations of these species and the prevalence of bTB is high. Additionally, mycobacteria can survive in the environment (e.g. water, pastures), which can thus become a potential source of infection [13].

**Fig. 2:**
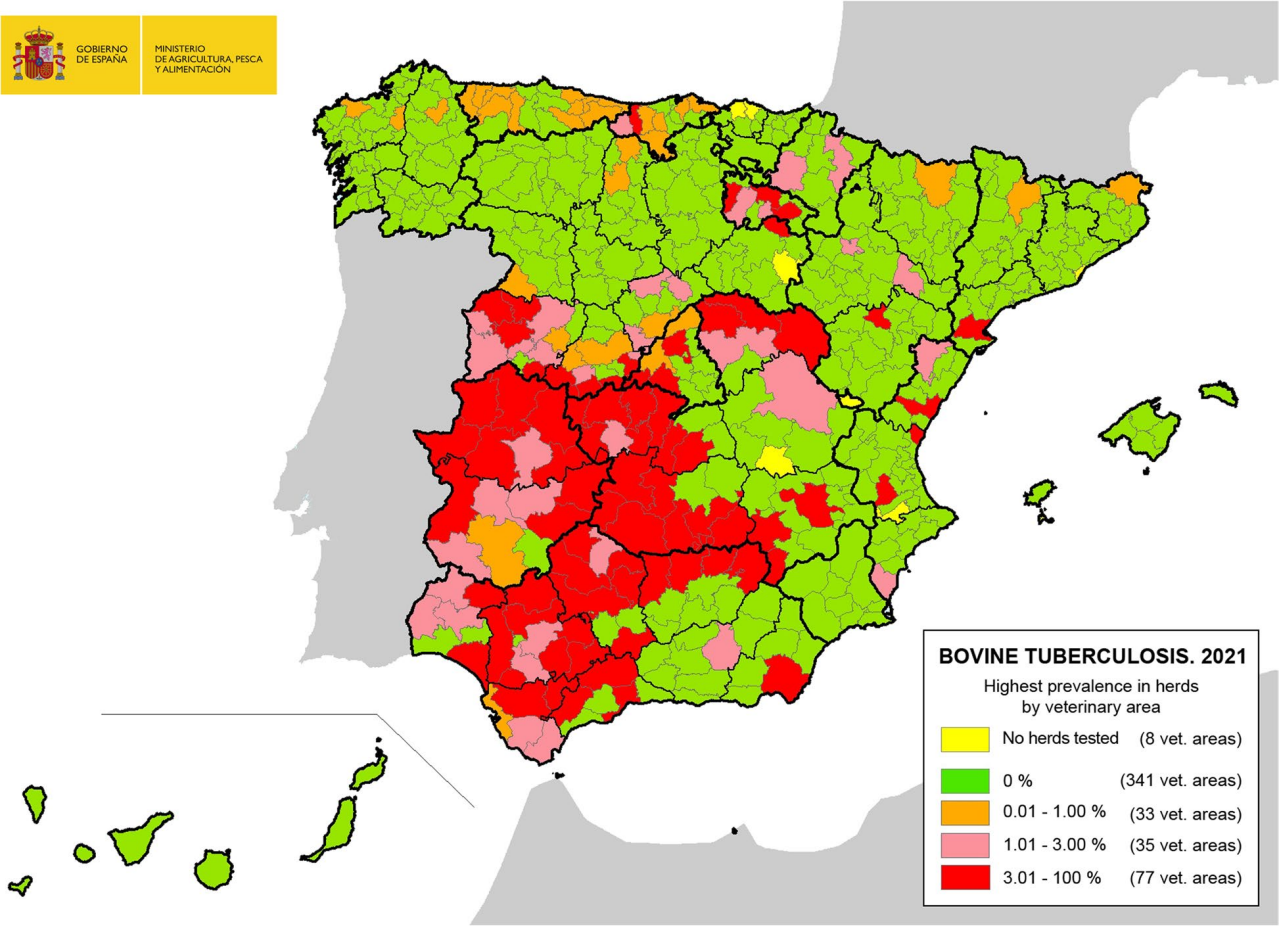
Herd prevalence of bovine tuberculosis in the different veterinary areas of Spain in 2021. (Data provided by the Spanish Ministry of Agriculture, Fisheries and Food, 2022)

The highest prevalence of bTB in Spain is in the west-central and southern regions (Fig. 2), where the largest populations of beef and bullfighting herds are located. In 2021, the regions with the highest prevalence of bTB infection (considered high prevalence regions, where prevalence is ≥ 1%) were Castilla-La Mancha (8.96), Andalucia (6.57), Extremadura (4.33), Madrid (2.76), Valencia (1.18) and Castilla y Leon (1.34), although this indicator has improved in most of them in the last three years (Table 1). Moreover, the presence of other domestic and wildlife reservoirs of TB has been reported in these regions, as has also occurred in other countries such as the Republic of Ireland, which complicates the epidemiology of the disease and can make control and eradication even more difficult [14].

**Table 1:** Herd incidence of bovine tuberculosis in the period 2003–2021 in the different Autonomous Communities of Spain (data provided by the Spanish Ministry of Agriculture, Fisheries and Food, 2022)

Region	Herd incidence																		
	2003	2004	2005	2006	2007	2008	2009	2010	2011	2012	2013	2014	2015	2016	2017	2018	2019	2020	2021
Andalucía	4.82	3.71	3.62	1.76	2.65	2.92	6.83	5.08	4.01	4.59	3.36	7.72	11.53	9.05	4.66	3.99	3.05	2.70	3.22
Aragón	0.94	0.71	1.47	1.27	2.92	0.30	0.61	0.59	1.06	0.72	0.39	0.15	0.61	0.43	0.39	0.22	0.25	0.25	0.09
Asturias	0.20	0.20	0.14	0.13	0.22	0.19	0.16	0.11	0.12	0.18	0.16	0.21	0.22	0.15	0.06	0.05	0.08	0.09	0.11
Baleares	1.02	0.65	0.65	0.00	0.00	0.00	0.00	0.17	0.00	0.40	0.40	0.41	0.20	0.00	0.00	0.31	0.00	0.00	0.00
Canarias	1.05	2.06	0.60	0.22	0.37	0.16	0.00	0.00	0.00	0.00	0.00	0.00	0.00	0.00	0.00	0.00	0.00	0.00	0.00
Cantabria	0.94	1.02	0.90	0.80	1.82	0.89	0.69	0.52	0.56	0.65	0.63	0.40	0.97	0.65	0.40	0.48	0.45	0.40	0.68
Castilla La Mancha	0.00	0.91	2.84	4.15	5.54	5.36	3.89	1.39	1.13	1.23	1.69	2.20	3.60	3.98	5.26	14.93	4.65	4.37	3.51
Castilla y León	3.42	2.56	2.41	3.19	3.35	2.24	1.34	1.82	1.77	1.83	2.04	1.31	1.20	1.50	1.26	1.08	1.06	0.20	1.10
Cataluña	1.47	1.35	1.23	0.85	0.52	0.33	0.44	0.44	0.47	0.12	0.02	0.14	0.20	0.16	0.18	0.06	0.04	0.04	0.06
Extremadura	0.58	2.88	2.04	0.62	0.68	1.47	2.11	1.27	1.90	2.22	3.02	2.69	10.26	6.52	4.69	4.81	3.27	3.24	2.76
Galicia	0.36	0.42	0.27	0.18	0.06	0.08	0.20	0.18	0.11	0.12	0.09	0.11	0.05	0.04	0.02	0.05	0.03	0.02	0.02
La Rioja	1.80	2.15	0.98	0.36	0.70	1.45	0.75	1.14	0.38	0.36	0.37	0.36	2.46	3.51	1.41	1.43	5.82	3.09	2.00
Madrid	0.87	1.12	1.42	1.87	2.62	4.27	2.91	3.20	5.51	3.60	2.37	2.48	1.86	1.33	1.20	1.68	1.48	1.70	1.89
Murcia	1.48	6.70	4.46	2.35	7.53	2.10	3.19	1.59	0.33	1.40	1.47	0.94	1.66	2.58	0.62	0.30	0.00	0.00	0.00
Navarra	0.43	0.26	0.38	0.22	0.28	0.23	0.24	0.61	0.42	0.12	0.48	0.25	0.19	0.51	0.41	0.39	0.19	0.32	0.19
País vasco	0.15	0.19	0.16	0.19	0.14	0.18	0.55	0.28	0.29	0.16	0.17	0.20	0.12	0.11	0.07	0.00	0.00	0.05	0.00
Valencia	0.89	0.66	0.59	1.25	1.14	1.41	1.03	2.01	0.88	1.36	2.40	2.70	2.14	1.79	3.79	2.60	1.50	0.69	1.18
Total	**1.06**	**1.11**	**0.99**	**0.84**	**1.02**	**0.87**	**1.03**	**0.85**	**0.84**	**0.90**	**0.91**	**1.06**	**2.02**	**1.59**	**1.15**	**1.35**	**0.92**	**0.75**	**0.90**

In order to monitor and control the distribution of MTBC members in animals in Spain, it is necessary to collect all the information of epidemiological interest, such as the geographical location, the animal species from which the microorganism was isolated or the genotype (spoligotype, VNTR profile, etc.). It is, therefore, essential to develop databases containing this information that users can consult and use in their epidemiological studies. The Spanish Ministry of Agriculture, Food and Fisheries (MAPA) has several databases. The REGA database (register of livestock establishments) is a multi-species register containing all the data concerning the establishments located in Spain. The REGA, REMO (register of movements) and RIIA (register of animal identifications) are essential parts of SITRAN, which is an integral traceability system. Moreover, in 2009, the BRUTUB database was created so as to include all the epidemiological surveys conducted on positive farms in order to allow their analysis and identify risk factors related to the occurrence of bTB outbreaks.

**Fig. 3:**
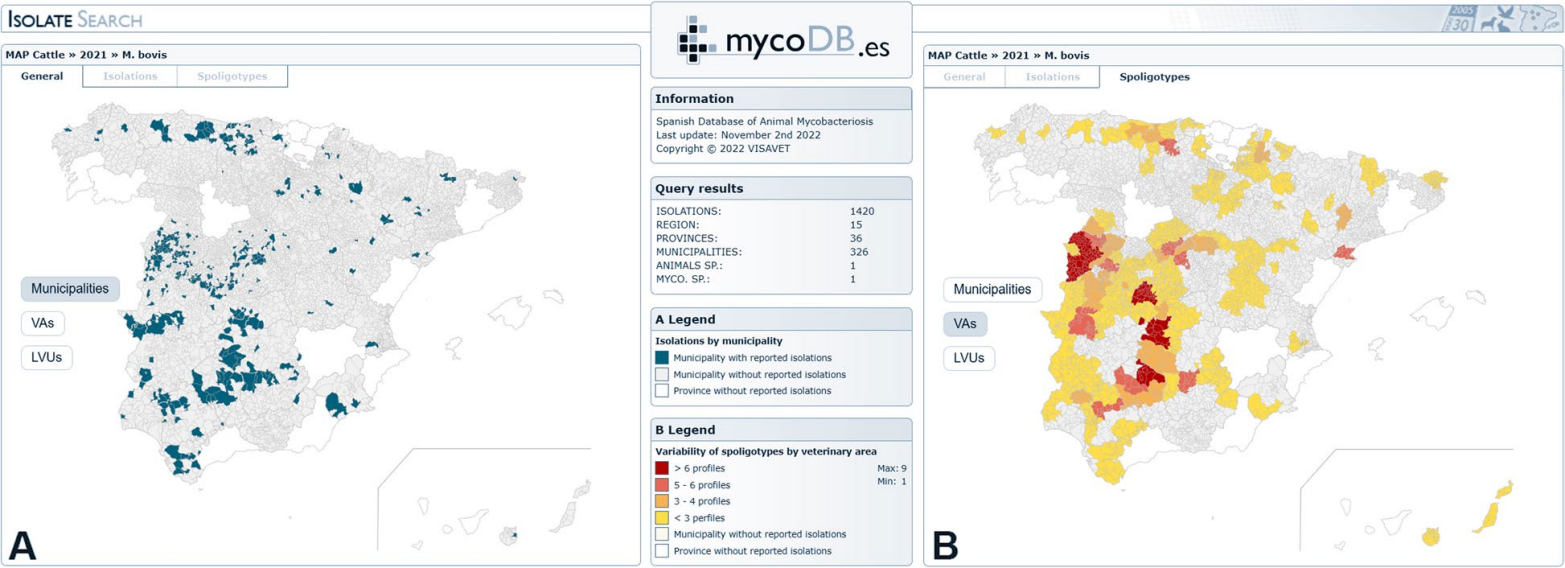
MycoDB.es database results of an isolate query of *Mycobacterium bovis* in cattle in 2021. **A** Isolations by municipality. **B** Variability of spoligotypes found in the different veterinary areas

Additionally, databases containing epidemiological and molecular data regarding the MTBC isolates recovered are also available. From the point of view of Animal Health, Mbovis.org (currently hosted bythe VISAVET Health Surveillance Center, VISAVET-UCM) is one of the most important international databases on animal TB [15]. Its main function is the assignment of an internationally standardised nomenclature for all MTBC isolates of animal origin. This database is freely accessible and includes maps of those countries reporting new spoligotypes. Other widely used databases, such as SITVIT2, SITVITbovis [16] or MIRU-VNTRplus [17], contain data on MTBC isolates mostly obtained from clinical cases in humans. In Spain, mycoDB.es is of paramount importance: this database was established in 2008 thanks to collaboration between the MAPA and VISAVET-UCM, and contains epidemiological and molecular data concerning all MTBC isolates (mainly *M. bovis* and *M. caprae*) obtained in Spain. These data include the animal species of origin, the geographical location, the year of isolation, the spoligotyping profile and the MIRU-VNTR profile (if available). The mycoDB.es database [18] contains records dating back to 1996, and currently includes more than 47,000 isolates, 564 spoligotypes and 182 MIRU-VNTR profiles. There are several search tools with which to customise the querying of the data, thus allowing the combination of several parameters, such as the animal species from which it was isolated, the autonomous community, province or municipality, the year of isolation, the species of MTBC and even the spoligotype. Moreover, these searches are presented by means of a geographical viewer on a map, thus facilitating their visualisation (Fig. 3). While Mbovis.org and the other databases are for public use, mycoDB.es can be accessed only by Spanish veterinary services and official laboratories that participate in the bovine TB eradication programme.

Transmission models have also been used in order to study TB infection in more specific conditions in Spain. Alvarez and collaborators estimated the coefficient of intra-herd transmission using data obtained from herds in the Autonomous Region of Madrid that included the main farming industries in Spain (beef, dairy and bullfighting) [19]. In this study, the authors demonstrated the influence of the productive type on transmission of the disease, since the estimated transmission coefficients (related to the expected number of animals newly infected by an infectious individual per unit of time – here, in years) in dairy herds (median estimate for β = 4.3 cattle/year) were significantly higher than in beef (median β = 2.3 cattle/year) and bullfighting (median β = 2 cattle/year) herds, possibly owing to the greater contact between animals under more intensive production conditions. This demonstrates the importance of the productive type in the recovery of the OTF status and, therefore, the need to incorporate such information into the construction of transmission models. Ciaravino and collaborators used a compartmentalised stochastic model to estimate the within-herd bTB transmission parameters and reported a median value for the transmission coefficient of 5.2cattle/year. When considering a frequency of routine controls of six months, the mean value of Rh (mean number of secondary cases caused by a single infected animal introduced into a herd) was 0.23, increasing to 0.82 when annual frequencies were considered [20].

The identification of both the origin of bTB outbreaks and the main factors that contribute to an increased risk of infection can provide important indications for the design of effective prevention and control strategies. According to the Spanish bTB eradication programme, for each newly infected herd confirmed by culture (i.e. a bTB breakdown), the official veterinary officers (VOs) identify the possible source of the breakdown by conducting a questionnaire-based epidemiological investigation. At the end of each questionnaire, the VOs should also indicate what, in their opinion, is the most likely cause of the breakdown. Since 2009, these data have been recorded in the BRUTUB database, which is held by MAPA. Furthermore, since 2012, epidemiological surveys have also been conducted on skin-test negative farms (i.e. control farms) selected by the VOs through pairing by herd size and location in order to identify potential risk factors, and the data obtained have been recorded in the BRUTUB database.

Guta and collaborators investigated the origin of the bTB breakdowns reported in Spain between 2009 and 2011 by analysing the data recorded in this database (22% of the total breakdowns). The investigation showed that the most frequent cause was residual infection, followed by interaction with wildlife reservoirs [21], results that were later confirmed in a different study [22]. Using this study and previous studies regarding the role of wildlife and the performance of diagnostic tests as a basis, the national eradication programme was reinforced through the implementation of measures such as compulsory training courses (since 2012) for both private veterinarians conducting bTB testing and VOs involved in the management of the disease, and the strengthening of audits of field testing practices.

In addition to these studies, great efforts have been made at national and regional levels to discern which other risk factors may shape the risk of occurrence and the duration of bTB outbreaks. In this respect, studies have been carried out in order to both analyse the movement of animals as a source of bTB [23] and identify risk factors associated with the duration of an outbreak, such as the prevalence of TB herds at the county level, herd size, number of animals entering the herd in the previous three years, number of skin test reactors in the initial positive test and number of days between this test and follow-up tests [24]. Finally, several epidemiological and scientific studies carried out in recent years have shown the important role that wild animals play in the transmission and maintenance of diseases shared among domestic livestock, wildlife and even humans, such as tuberculosis. Taking into account that the interaction with wildlife can be a source of infection, a surveillance programme for wildlife (Plan de Actuaciónsobre Tuberculosis en Especies Silvestre, PATUBES) was implemented in 2017 in order to further reduce the occurrence of bTB breakdowns [5]. The main objectives of this programme are the surveillance and control of other domestic animals and wildlife, the classification of Spanish regions depending on the risk and the establishment of specific measures such as the elimination of hunting byproducts, density controls, training and biosecurity.

All the information obtained from epidemiological studies is valuable for stakeholders and its implementation in future strategies in eradication and control programmes.

## Bovine tuberculosis eradication programme for Spain

The first actions taken against bTB began in Spain in the early 1950s. In 1965, a national plan to control and eradicate bTB and brucellosis was established, focused mainly on the dairy cattle population located in the north and centre of Spain. After the inclusion of Spain in the EEC, Spain presented an Accelerated Eradication Programme in 1987, in accordance with Directives 77/391/EEC and 78/52/EEC and Decision 87/58/EEC. Efforts have since been made to apply the eradication campaign in a homogeneous manner throughout Spain and in all production types (dairy since 1965, beef since 1993 and bullfighting since 2004).

The National Programmes for the Eradication of Bovine Tuberculosis 2006–2010 represented a qualitative change in the approach employed to attain the objectives, since they laid down the principles required to guarantee continuous actions over time using a multiannual approach, established over a 5–year period. One of the main objectives of these programmes was the gradually increase in diagnostic sensitivity (Se) at both herd and individual levels. Moreover, specific measures based on previous studies were gradually implemented, such as the control of wildlife reservoirs or the effective implementation of a post-mortem surveillance system in slaughterhouses. In fact, up to 8–9% of all bTB cases in Spain are detected as the result of the passive surveillance at the slaughterhouse, whereas the remaining cases are detected as the result of the active component of the eradication programme (personal communication, MAPA). However, according to a recent study carried out in a region of Spain, the probability of detecting animals with bTB lesions is highly influenced by the slaughterhouse at which an animal is slaughtered, ranging from 603 to 3,070 per 10,000 animals for the reactors and 0.2–16.1 per 10,000 animals for the non-reactors [25]. In addition to the animal subpopulation defined by the results obtained in the bTB diagnostic tests (reactor/non-reactor), the production type of the farm of origin and its size, the breed and age of the animal, the year and also the season in which an animal was slaughtered influence the probability of detecting lesions in slaughtered animals.

The official diagnostic tests used are those established by Royal Decree 2611/1996, Sect. 2 of Annex III (diagnostic methods for the granting and maintenance of disease-free status) and article 9 (classification of suspected cases and confirmed cases by histopathology, immuno-histochemistry, direct PCR from tissue samples and bacteriology) of Regulation (EU) 2020/689 and the National Eradication Programme. Ante-mortem diagnostic tests are carried out in animals older than 6 weeks in the case of the single and comparative intradermal tuberculin (SIT and CIT) tests and 6 months in the case of the gamma-interferon release assay (IGRA), which are typically used in parallel with the intradermal test in confirmed bTB-infected herds. The frequency of tests is established according to the prevalence in the different areas and is specified in the National Eradication Programme. The IGRA is carried out by laboratories authorised in accordance with the aforementioned Royal Decree 2611/1996, which periodically participate in inter-laboratorial tests carried out by the Spanish NRL. Although the use of the IGRA can help to overcome some of the disadvantages associated with the use of SIT/CIT tests, its use as a stand-alone test is limited owing to its comparatively lower diagnostic specificity (Sp). The fact that different commercial kits are available signifies that a careful assessment of the performance of these kits help to adjust the cut-off values on the basis of the strategy of use and the objectives [26]. In Spain, the positive impact of the use of IGRA has been demonstrated owing to a decrease in the time required to restore the officially bTB free status in infected herds when compared with situations in which it is not used [19].

The routine technique employed in order to obtain and maintain the officially bTB free status is mainly the SIT test, although the CIT test can also be used under specific epidemiological circumstances and at the discretion of the competent authority. Since Regulation (EU) 2016/429 came into force, the IGRA test can be used as an alternative to the SIT/CIT tests in animals older than 6 months, always at the discretion of the competent authority, as a routine test by which to grant or maintain the OTF status and as a pre-movement test, in accordance with Sect. 2 of Annex III of Regulation (EU) 2020/689 and the protocol published for this purpose on the European Union Reference Laboratory (EU-RL) website, in accordance with Article 6 of Regulation (EU) 2020/689. In this respect, the scientific opinion published by the EFSA regarding the suitability of the IGRA for inclusion as an official primary or stand-alone test and as an equivalent to the intradermal test used to define the infectious status [27] has been of paramount importance for the recent inclusion of the IGRA test (EU-RL protocol) as a stand alone test for specific purposes (granting and retaining official TB-free herd status and for certification for intra-European Union trade of bovine animals) specified in Regulation (EU) 2016/429.

Nevertheless, the EFSA employed previous studies as the basis on which to conclude that the Sp of the IGRA might not, in certain conditions, be as high as that of the SIT test, which is the skin test with the lowest Sp approved in the EU. Therefore, and taking into consideration ongoing research on improved antigens, the EFSA recommended further evaluations of the performance of newly available specific antigens for blood stimulation in the IGRA with the aim of improving its Sp [27]. In this respect, a recent antigenic cocktail developed and evaluated in a study by Middleton and collaborators showed that increasing the Sp of the IGRA (maintaining a similar Se) is possible by using defined antigens [28]. Nevertheless, further studies with a large number of animals and in a wide range of different epidemiological situations are neccessary in order to confirm these findings.

Several studies have been carried out in order to evaluate the performance of the official diagnostic tests (ante-mortem and post-mortem) and the overall effectiveness of the bTB diagnostic strategy in Spain [29, 30], and results from these studies have allowed the further improvement of the bTB eradication programme in Spain, leading to a decrease in prevalence in recent years and to the achievement of the OTF status in several regions.

The eradication programme in Spain allows the use of strategies depending on bTB prevalence: in OTF regions (A) or regions with 0 prevalence (the Canary Islands, Galicia, the Basque Country, Asturias, Catalonia, Murcia and the Balearic Islands), the competent authority may extend the interval between routine herd-tests from one to two years. In addition, in regions that have maintained a herd prevalence of below 1% (low prevalence regions) for two consecutive years (B) the interval between the routine herd-tests may be extended to two years (24 months) in historically TB-free (hOTF) establishments (with a previous risk analysis). This occurs in provinces in which 100% of the target herds are included in the national programme and specific herds such as bullfighting herds or herds using shared pastures are excluded. In (non-bullfighting) hOTF establishments, SIT or CIT tests can be used as a routine test on the basis of a previous risk assessment. If positive animals are detected by using the CIT test, the results should be re-evaluated on the basis of SIT. The IGRA protocol published by the EU-RL can also be used as a routine test with the same considerations as those of the SIT test. Similarly, if the competent authority decides to apply the CIT test, those animals that attain a positive result in the single interpretation (increase in the skin fold thickness greater than or equal to 4 mm to the bovine PPD, without clinical signs) but a negative result in the CIT test (owing to a larger increase in the skin fold thickness on the avian PPD inoculation site) are considered “follow-up cattle”. Their movements are subsequently restricted and they are subjected to post-mortem sampling when sent to the slaughterhouse or in the by-product processing plant in order to carry out bacteriology. SIT or IGRA tests are used for the routine testing of the remaining OTF herds. Non-OTF herds must be tested at least twice a year. Finally, in regions with a bTB prevalence higher than 1% (high prevalence regions), the control measures are more severe and the SIT and IGRA tests are used for routine testing even in OTF herds (excluding specific cases distinguished on the basis of previous epidemiological studies).

In Spain, the measures adopted in order to deal with positive cases are described in Chapter II of Royal Decree 2611/1996 and in articles 24 to 31 of Regulation (EU) 2020/689. Particular emphasis is placed on those establishments in which a positive animal has been detected following the prophylactic measures established in article 24 of Royal Decree 2611/1996. Specifically, the OVs will certify that the cleaning and disinfection measures have been correctly carried out, that a minimum period of 60 days of sanitary vacuum for the reuse of pastures has been applied, and that manure has been correctly managed. These certificates may be issued on the basis of others that have been completed by accredited and duly approved companies, mainly with regard to the management of manure and the disinfection of the facilities.

As described previously, the actions related to the control of TB in wildlife included in the National Eradication Programme were complemented with PATUBES, agreed at the central level with the other official authorities with competences in matters related to the environment and hunting and with the competent authority in matters regarding public health and food safety. This is in accordance with the recommendations made by the European Commission and anticipates the future European Legislation on animal health by incorporating wild species into the control of diseases that affect domestic livestock. The materialisation of PATUBES took place in Royal Decree 138/2020, as a basic legislative instrument that aims to control the risk to public health, animal health and the environment by reducing the spread of the disease among the different susceptible species of domestic and wild animals or those that are hunted.

Finally, it is important to highlight that the cornerstone of all bTB eradication programmes comprises the tuberculin tests, which are based on the intradermal injection of a reagent (purified protein derivative of *M. bovis*, bovine PPD tuberculin) developed at the end of the 19th century by Koch and initially conceived in order to cure the disease [14]. The intradermal tuberculin test continues to be the most important diagnostic tool, and the PPD tuberculin is one of the reagents most frequently used in livestock. Nevertheless, the composition of tuberculin is still poorly characterised, and there are significant differences regarding the potency of tuberculins produced by different manufacturers [31]. Moreover, the potency testing of the tuberculins is difficult and the process is not well standardised, requiring high costs and logistical demands. In this respect, the European Commission-DG Health and Consumers’s commitment to the standardisation of tuberculins is laudable. The composition of the bovine and avian PPDs used in Spain (CZV, Spain) were characterised by Infantes-Lorenzo and collaborators [32]: a total of 456 and 1019 proteins were identified in the bovine and avian PPD, respectively, 146 of which were shared by the two, which constitutes a potential source of cross-reactivity. In addition to factors associated with the current PPDs (such as their potency), other factors have been identified as potential causes of false negative and positive results to the intradermal tuberculin test in cattle, including factors related to the animals (early infection, anergy, concurrent immunosuppression), to the methodology (the inoculation dosage, the injection site, the lack of experience of the person administering the test) or even fraudulent activities [33–37]. The tuberculin potency of all batches should be evaluated by the NRLs (the Spanish NRL is located in Santa Fe, Granada), since it is vital to the outcome of the intradermal test: a significant difference in the number of reactors detected using high and low potency tuberculins has been reported [38]. The production of PPD tuberculins is standardised and regulated by the EU, since manufactures must fulfil the Good Manufacturing Practice conditions and comply with the European Pharmacopeia and WOAH requirements. The protein content of the tuberculins is not correlated with their biological activity and assays concerning the potency of tuberculin batches must, therefore, be performed in guinea pigs and cattle [39]. The requirement to check the potency in the bovine bio-assay was included in the old Directive 64/432/EEC and was recommended in the WOAH technical reports. However, this requirement was modified and removed from the Directive in 2002, mainly as a result of the high costs and logistical demands of the assays [14]. Potency tests are now rarely conducted in cattle and few laboratories, including the EU-RL for Bovine TB, carry out potency tests of bovine PPDs from different manufacturers in both guinea pigs and cattle.

## Future perspectives

The control and eradication of TB is a perfect example of the practical implementation of a One Health approach. In the case of animal TB, it is essential to include all the domestic and wildlife reservoirs together with the active involvement of all the stakeholders in order to achieve the objective of eradication (to be declared a TB-free Member State), which in the case of Spain and has been established for 2030. The objective is not easy, but the great advances made in recent years in aspects related to the epidemiology, diagnosis and control of the disease foster our optimism. In this respect, it is noteworthy that several regions of Spain have already attained this objective and that bTB surveillance is improving in the rest of Spain. Despite this, tuberculosis research remains essential, particularly as regards antemortem diagnosis, which is still a great challenge. Moreover, the consideration of sociological aspects in the eradication programme has gained relevance in recent years, especially with regard to the motivation of the veterinarians involved and to the improvements made to communication with the farmers in order to help them to realise they are an essential part of the eradication process and for them to appreciate the potential benefits in the medium and long term. There is currently a consensus among researchers and official veterinary services as to the feasibility of the objective established for 2030 if the positive evolution in the indicators of the programmes is maintained and the numerous advances in research continue to be applied.

## Data Availability

Not applicable.

## Abbreviations

bTB: Bovine tuberculosis
CIT: Comparative intradermal tuberculin
ECDC: European Centre for Disease Prevention and Control
EEC: European Economic Community
EFSA: European Food Safety Authority
EU: European Union
EU-RL: European Union Reference Laboratory
IGRA: Gamma-interferon release assay
MAPA: Spanish Ministry of Agriculture, Fisheries and Food
MS: Member State
MTBC: *Mycobacterium tuberculosis *complex
OTF: Officially Tuberculosis Free
hOTF: Historically TB-free
PATUBES: Plan de Actuación sobre Tuberculosis en Especies Silvestre
PPD: Purified protein derivative
Se: Diagnostic sensitivity
SIT: Single intradermal tuberculin
Sp: Diagnostic specificity
VO: Official veterinary officer
WHO: World Health Organisation
WOAH: World Organization for Animal Health
zTB: Zoonotic tuberculosis
